# Prostate Artery Embolization vs. Holmium Laser Enucleation of the Prostate: A Matched Pair Analysis of Functional Outcomes and Complications

**DOI:** 10.3390/jcm14248906

**Published:** 2025-12-16

**Authors:** Simon Hannes Friedrich Leschik, Robert Große Siemer, Friedrich-Carl von Rundstedt, Philipp Gild, Christian P. Meyer, Raisa S. Abrams-Pompe, Ulf Teichgraeber, Thomas Lehmann, Susan Foller, Marc-Oliver Grimm, Tobias Franiel

**Affiliations:** 1 University Hospital Jena, Friedrich Schiller University, 07743 Jena, Germany; 2 Department for Urology, Helios University Hospital Wuppertal, University Witten-Herdecke, 58455 Wuppertal, Germany; 3 Department of Urology, University Medical Center Hamburg-Eppendorf, 20251 Hamburg, Germany; 4 Department of Urology, Ruhr University Bochum, Campus OWL, 32049 Herford, Germany; 5 Institute of Diagnostic and Interventional Radiology, Friedrich Schiller University, 07743 Jena, Germany; 6 Center for Clinical Studies, Friedrich Schiller University, 07743 Jena, Germany; 7 Department of Urology, Friedrich Schiller University, 07743 Jena, Germany

**Keywords:** benign prostatic hyperplasia, lower urinary tract symptoms, HoLEP, PAE, complications, erectile function, matched-pair analysis

## Abstract

**Background**: This retrospective matched-pair analysis compared functional outcomes and complications of prostate artery embolization (PAE) using 250 µm microparticles and holmium laser enucleation of the prostate (HoLEP) in patients with lower urinary tract symptoms (LUTS) due to benign prostatic hyperplasia (BPH). **Methods**: A total of 69 PAE patients were matched 1:1 to 69 HoLEP patients using propensity scores based on age, prostate volume (PV), and IPSS. Follow-up was standardized at six months for the PAE cohort, while HoLEP outcomes were assessed cross-sectionally (median 52.9 months). All comparisons were therefore interpreted as cross-sectional analyses rather than time-matched outcomes. Secondary endpoints were complications according to the Clavien–Dindo Classification. **Results**: At baseline, there were no significant differences between PAE and HoLEP regarding IPSS, QoL, or Qmax. Both interventions led to significant within-group improvements in IPSS, QoL, and Qmax (*p* < 0.001). Between-group comparisons demonstrated significantly greater improvement in IPSS, Qmax, and QoL following HoLEP (all *p* < 0.05). Erectile function remained stable after PAE and showed a non-significant decrease after HoLEP. Severe complications (Clavien–Dindo ≥ Grade III) were not observed after PAE. These findings should be interpreted considering the study’s main limitations, including the small cohort size, its retrospective matched-pair design, and variability in surgeons’ HoLEP experience. **Conclusions:** PAE with 250 µm microparticles and HoLEP are both effective and safe procedures. While PAE compared to HoLEP is less effective regarding functional outcome, it showed no difference in QoL improvement and is associated with no greater grade II complications.

## 1. Introduction

LUTS and BPH are common in older men, and incidence increases with age [1]. In most cases, LUTS is related to BOO caused by BPH, and therefore the primary goal is an effective symptom relief [2]. First-line treatment consists of lifestyle interventions and medication like alpha-adrenoreceptor antagonists and can be supplemented by 5α-reductase inhibitors. The treatment should always consider the size of the prostate. If symptoms persist or progress, surgical therapy is generally needed. Various surgical interventions have emerged as effective options, each with its unique advantages and considerations. In addition to established transurethral procedures such as TURP, several minimally invasive techniques, including prostatic urethral lift, convective water vapor energy ablation, and bipolar or thulium laser vaporization, have broadened the therapeutic armamentarium for LUTS and BPH. These approaches aim to reduce perioperative morbidity and shorten recovery while maintaining adequate symptom relief in selected patient groups. Within this expanding field, two such techniques, PAE and HoLEP, have gained prominence for their efficacy in alleviating BPH-related symptoms.

Recent guideline trends emphasize the importance of offering both surgical and minimally invasive options that can address BOO across different prostate sizes and comorbidity profiles. A recent review also highlighted that delayed treatment of BPH may contribute to irreversible bladder dysfunction and that preservation of sexual function has become an increasingly important factor in treatment selection, supporting a broader integration of minimally invasive techniques in appropriately selected, sexually active patients [3]. This consideration is particularly relevant since functional domains such as continence and sexual function increasingly influence therapeutic decision-making. In this context, HoLEP and PAE have attracted particular attention because both techniques provide volume-independent applicability and are increasingly available across tertiary centers. As their clinical roles continue to expand, a clear delineation of the therapeutic boundaries between HoLEP and PAE is lacking, and real-world comparative data remain limited. Therefore, studies directly comparing PAE and HoLEP are needed to better define patient selection, clarify expected functional outcomes, and support evidence-based clinical decision-making.

HoLEP is an established surgical treatment for BPH, particularly in patients with large prostates, and is increasingly used worldwide.

Unlike TURP, HoLEP is effective across all prostate sizes and is associated with lower bleeding risk, especially in patients receiving anticoagulation therapy [4,5]. Current EAU guidelines recommend treatment with HoLEP as a size-independent endoscopic surgical alternative to TURP or open surgery of the prostate [6].

A recent study has identified PAE as a developing alternative to surgical treatment of BPH [7]. In 1979, Lang et al. were the first to describe PAE as an efficient treatment for hemorrhage from the bladder and other pelvic organs [8]. The first intentional treatment of BPH using PAE was described nearly thirty years later [9]. Since then, just a few studies have evaluated PAE regarding efficiency and safety. In 2018 Abt et al. compared PAE to TURP in a randomized trial in order to identify differences in functional outcomes and complications [10]. In this study, prostate volume was measured by transabdominal ultrasound (PAE 51.2 ± 16.5 mL vs. HoLEP 52.1 ± 18.6 mL) and by magnetic resonance imaging (PAE 52.8 ± 32.0 mL vs. HoLEP 56.5 ± 31.1 mL). PAE was inferior in functional outcomes but showed fewer complications, whereas HoLEP is a safe, highly efficacious, and size-independent procedure [11].

Correct indications for PAE are still in scientific evaluation. Although PAE and HoLEP are increasingly used in clinical practice, direct comparative data between these two volume-independent interventions remain limited. Existing studies have primarily examined PAE in comparison to TURP, while HoLEP has been evaluated mostly against other endoscopic techniques. Consequently, evidence on how PAE performs relative to HoLEP, particularly regarding functional outcomes and complications, is sparse. Furthermore, both procedures target partially overlapping patient populations, raising important questions about optimal treatment selection in men with moderate-to-large prostates. A comparison therefore provides clinically relevant insight into treatment pathways and may help define thresholds at which one modality becomes preferable over the other. In this context, the purpose of this study was to identify differences between PAE and HoLEP in prostates > 30 mL.

## 2. Methods

### 2.1. Study Population

In this retrospective matched-pair study, we included 138 patients undergoing either PAE or HoLEP (69 patients each) due to symptomatic BPH with LUTS. All patients undergoing PAE were treated at BLINDED between 2014 and 2017 as previously described in BLINDED [12]. Inclusion criteria for the PAE cohort were age > 45 years, a prostate volume > 30 mL, an IPSS ≥ 18, and a Qmax ≤ 15 mL/s or supply with a transurethral catheter. All patients gave informed consent to the study that was approved by the local ethics committee.

HoLEP patients were treated between 2010 and 2016 in the BLINDED and were acquired from an institutional database as previously described by BLINDED [13]. This existing institutional surgical database routinely collects information on all endoscopic prostate procedures. These data were retrospectively extracted and included baseline characteristics, perioperative parameters, and available follow-up information. Patients from HoLEP had either a urodynamically proven obstruction or clinical signs suggesting LUTS due to BPH. All data were fully anonymized.

### 2.2. Rationale for Matching and Study Design

The two procedures compared in this study differ fundamentally in their approach, invasiveness, and typical patient selection in clinical practice. A matched-pair analysis was therefore chosen to reduce baseline differences that could otherwise bias the comparison. Matching was performed based on age, prostate volume, and IPSS.

A randomized design was not feasible due to the retrospective nature of the available data. Using an existing institutional surgical database for HoLEP and a prospectively collected PAE cohort allowed us to analyze a clinically relevant real-world population but also made matching necessary to achieve a comparable starting point between groups. Despite matching, PSA and IIEF remained imbalanced.

### 2.3. General Assessment

Validated questionnaires (IPSS, QoL, and the IIEF) were used to determine symptoms at baseline and follow-up. Furthermore, all patients underwent a general and standard urologic preoperative evaluation, including clinical history, DRE, blood and urine analysis, PSA, Qmax, and post-void residual volume by transabdominal ultrasound. Follow-up in the PAE cohort was performed longitudinally at 1, 3, and 6 months. For statistical analysis the 6-month data were used. In the HoLEP group, follow-up data were collected cross-sectionally and did not occur at a predefined time point. The median time between surgery and follow-up was 52.9 months (IQR: 35.5–81.7 months) in the latter cohort. As no correlation could be established between the follow-up data or with the outcomes, it was decided to forgo this analysis. Postoperative complications were recorded and assessed according to Clavien–Dindo Classification within the hospital stay and up to thirty days afterwards. Incontinent patients were defined as those who required the use of more than two absorbent pads per day postoperatively.

### 2.4. Interventional Procedure

All PAE patients were assessed by PSA and MRI to rule out risk for significant prostate cancer before the PAE procedure. Suspicious cases underwent a transrectal biopsy beforehand. PAE was performed using a bilateral or at least unilateral embolization after catheterization of prostatic arteries using spherical, Polyzene F-coated hydrogel microspheres with a size of 250 µm (Embozene; Boston Scientific, Marlborough, MA, USA). Clinical workflow included perioperative antibiotics and continuing BPH medication for one month. The bladder catheter, which all patients received as part of the intervention, was removed on the same day.

### 2.5. Surgical Procedure

All prostates were enucleated in a one-, two-, or three-lobe technique by using a holmium 100 W (Lumenis, Yokneam, Israel) laser as described in a previous publication [13]. Perioperative management included prophylactic antibiotics based on local resistance patterns or preoperative urine culture results. The removed tissue was histopathologically evaluated by a pathologist. Postoperatively, the patient received a 22F bladder catheter for at least 48 h. After catheter removal, uroflowmetry was measured. Likewise, perioperative antibiotics were administered either according to protocol or according to preoperative urine culture.

### 2.6. Endpoints

The primary endpoints of interest encompassed a comprehensive evaluation of patient outcomes, comprising IPSS, QoL assessments, peak urinary flow measurements, and IIEF. These endpoints were meticulously chosen to provide a thorough understanding of the therapeutic impact on urinary function, overall well-being, and sexual health.

Additionally, a spectrum of secondary endpoints was explored, focusing on post-intervention complications as classified by the established Clavien–Dindo Classification. This comprehensive approach enabled us to gain insights into the safety profiles of both interventions, offering a nuanced assessment of their respective clinical effectiveness and patient tolerability.

### 2.7. Statistical Analysis

Based on 69 available PAE patients, 69 HoLEP patients (out of a collective of 1800 patients from an institutional database) were matched using propensity scores (nearest neighbor) in a 1:1 fashion. Variables used in the regression model were IPSS, prostate volume, and age. Descriptive statistics were depicted as median and interquartile range (IQR), and significance between values was evaluated either by Mann–Whitney-U test or *t*-test. A *p*-value of <0.05 was considered statistically significant. Changes in IPSS, QoL, Qmax, and IIEF between follow-up and baseline were compared between the PAE and HoLEP groups due to the matched design by the two-sided Wilcoxon test. Hodges–Lehmann (HL) estimates were provided with 95% confidence intervals (CI) to assess the group difference in these changes. The absolute number of complications was counted, and frequency was calculated in each subgroup. Further statistical and graphical analysis was performed by using SPSS (Version 28).

### 2.8. Baseline Characteristics

The baseline characteristics of both subgroups are shown in Table 1. The median age was 66 years (62–71) in HoLEP and 67 years in PAE (58–72). Both groups were well balanced regarding baseline characteristics, though we noted two significant differences in IIEF and PSA between both groups (*p* < 0.05). In the HoLEP group, the PSA median was 4.9 ng/mL, whereas in the PAE group, it was 2.4 ng/mL (*p* = 0.001). Additionally, the IIEF median was 18.5 in the HoLEP group and 23 in the PAE group (*p* = 0.002).

## 3. Functional Results

**Figure 1:**
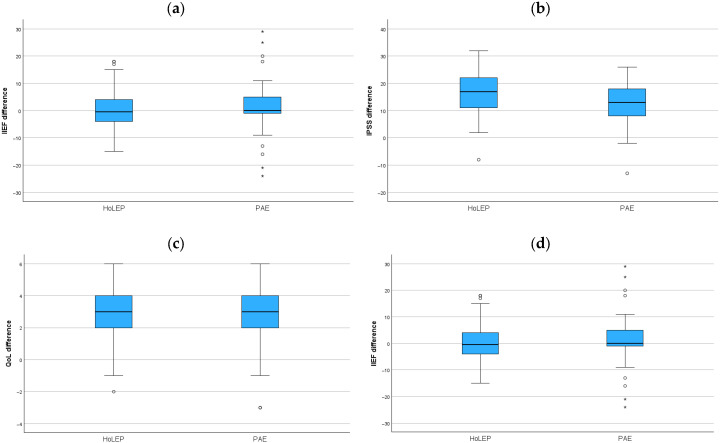
(**a**–**d**) Post-interventional differences in IIEF (**a**), IPSS (**b**), QoL (**c**), and Qmax (**d**) for HoLEP vs. PAE. Significant differences were observed for IPSS ((**b**), *p* < 0.001), QoL ((**c**), *p* < 0.001), and Qmax ((**d**), *p* < 0.001), all favoring HoLEP. The difference in IIEF was not statistically significant in either group (HoLEP: *p* = 0.7; PAE: *p* = 0.2). Circles indicate outliners and asterisks indicate extreme outliners.

All functional parameters pre- and postoperatively are depicted in Table 2. In comparison to baseline characteristics, IPSS, QoL, and Qmax showed clinical improvement in both groups after treatment to the time of follow-up. Both interventions resulted in comparable QoL improvements, each reaching a median score of 3 (2–4) (*p* < 0.001). PAE patients demonstrated a Qmax improvement of 5.8 mL/s (2.2–9.2) versus 14.9 mL/s (10.2–25.9) in the HoLEP group (*p* < 0.001). Both groups showed a significant improvement in IPSS at their respective follow-up (HoLEP: 23 (16.5–27) vs. 4 (1–8.5), *p* < 0.001; PAE: 23 (17.5–26) vs. 8 (5–13), *p* < 0.001). As shown in Figure 1, the post-interventional IPSS was significantly improved after HoLEP by 17 (10.5–22) and 13 (8–18.5) after PAE (*p* < 0.001). The preoperative median IIEF of 23 (9.5–26) in the PAE group increased slightly compared to the postoperative median IIEF of 24 (10–29.5), although this difference was not statistically significant, with *p* = 0.2. In contrast, in the HoLEP group the preoperative median IIEF decreased from 18.5 (6.0–21) to 15 (8–23) postoperatively, although this was again statistically not significant different (*p* = 0.9).

The change in Qmax was significantly higher in the HoLEP group than in the PAE group (HL estimate 10.5, 95% CI: 4.4 to 16.7, *p* = 0.003), and there was also a significant higher decrease in the IPSS Score in the HoLEP group compared to the PAE group (HL estimate 3.5, 95% CI: 0.5 to 6.5, *p* = 0.019).

There were no significant differences in the changes in QoL (*p* = 0.332) and IIEF (*p* = 0.708) between both groups.

### Complications

All complications are listed in Table 3. Within the observation period, the majority of recorded complications were grade I complications according to the Clavien–Dindo classification. After PAE, four patients (5.8%) experienced a bleeding episode (either urethral or rectal) as a grade I complication, whereas postoperative pain was most common (8.7%) among HoLEP patients. Urinary infection as a grade II complication was observed in the HoLEP group in five (7.2%) cases versus one (1.4%) case in the PAE group, as well as a postoperative bladder tamponade associated with prolonged hospitalization (1.4%) and testicular infarction (1.4%), which both occurred in the PAE group only. More severe grade IIIa complications occurred in two patients (2.9%) after HoLEP due to postoperative bladder tamponade requiring surgical evacuation (1.4%) and the need for reinsertion of a urinary catheter (1.4%). Additionally, one grade IIIb complication (1.4%), postoperative severe bleeding, occurred in the HoLEP group. No grade IIIa or IIIb complications were observed in the PAE group. Given the limited sample size, the study may not have been powered to detect rare or high-grade (Clavien–Dindo ≥ III) complications, and the true incidence of such events should be interpreted with caution. During the database observation period, three patients died 6 and 7 years after HoLEP. The causes of death were pneumonia-associated sepsis, pancreatic carcinoma, and prostate carcinoma. As these were neither related to the hospital stay nor in temporal or causal connection, these complications were excluded from the analysis.

## 4. Discussion

While TURP has been considered to be the gold standard in BPH surgery for decades, the introduction of laser techniques has made even large glands amenable to transurethral treatments. Moreover, current evidence suggests a more personalized approach to BOO, including minimally invasive procedures like PAE [5,14]. In a landmark study Abt et al. compared PAE to TURP in 103 patients with a smaller BPH size (mean 50 mL) and found similar results with regard to patient-reported outcomes (IPSS) and inferior effectiveness concerning functional parameters [10]. These findings have now been supported by newly published long-term data from the same study, confirming that PAE provides sustained symptomatic relief in appropriately selected patients, although TURP remains superior in terms of objective functional outcomes over a 5-year period [15]. In addition, our results hold some important contributions for the comparison of PAE with HoLEP.

Maximum urinary flow and IPSS significantly improved in both groups, but improvements tended to be greater following HoLEP.

Earlier studies have suggested that PAE may help preserve erectile function in men with BPH, although the evidence remains heterogeneous and predominantly observational. Several investigations also reported improvements in quality of life following PAE [16]. In our data, erectile function remained stable after PAE and showed a slight, non-significant decrease after HoLEP.

Given the absence of statistical significance in both groups, this pattern should not be interpreted as preservation of erectile function by PAE but merely as a descriptive trend.

Indications and contraindications for PAE have been previously described in a review [7]. The results of the current study support the effectiveness and safety of PAE in large prostates (>60 mL) [17]. PAE is considered to be a mild treatment with fewer major complications [18,19]. Most patients in our PAE cohort just had minor grade I complications like transient inguinal hematoma or non-severe rectal bleeding. Recent evaluations have found that mis-embolization and other major complications occur in fewer than 3% of cases, reinforcing the favorable safety profile of contemporary PAE practice [20]. Nevertheless, in one case a testicular infarction occurred, and the patient needed an intravenous antibiosis and a suprapubic catheterization. It should be mentioned that since 2018, PAE at BLINDED has been performed with larger 400 µm sized microparticles instead of 250 µm microparticles. Since this time no testicular infarction or other with ≥ grade II misembolization occurred. Furthermore, one patient in our study experienced a PES with fever, leukocytosis, and a prolonged clinical stay after PAE. PES is seen as a main side effect of PAE, but incidence differs in the literature because of a lack of uniformity in the definition of symptoms according to Svarc et al. [21]. From our point of view, a severe PES due to PAE is a rare major complication.

Most of our HoLEP patients experienced just some minor postoperative complications like urinary infection and postoperative pain as described by other colleagues as well [22]. The postoperative catheterization rate of 1.4% is very low in our collective compared to other studies, in which this rate ranges from 2.9 to 7.9% [23]. In an analysis published by BLINDED with more than 1.800 HoLEP cases from our cooperating institute, the rate of failing to void after HoLEP differs between 4.5% and 6.5% according to age [13]. These differences might be caused by a selection bias due to our study design. Nevertheless, in comparison, the catheterization rate is higher in the PAE group in our study, maybe because of less effective functional outcomes by PAE.

Our study has three major limitations:

1. The limited cohort size inherently constrained the matching process and the statistical power of the study. Because only a fixed number of suitable matches were available, the inclusion of additional baseline variables in the propensity score model was not feasible without losing a substantial proportion of cases. Although PSA and IIEF differed between groups at baseline, these variables were therefore not included in the matching algorithm. PSA was omitted because it is not directly associated with LUTS severity or functional outcome parameters (IPSS, Qmax, QoL, IIEF) and was unlikely to confound the primary analyses. IIEF, despite its clinical relevance, could not be incorporated because no matching configuration produced adequate balance while preserving a sufficient sample size. Consequently, the baseline imbalance in IIEF must be acknowledged as a major limitation. Postoperative changes in IIEF were small in both groups, reducing the risk of significant bias in the comparison of erectile-function outcomes. Furthermore, no formal power calculation was performed. A meaningful a priori power analysis would have required predefined effect sizes and the ability to increase the sample accordingly; however, this was not possible in a retrospective matched-pair design with a fixed number of eligible controls. Thus, the study is not powered to detect minimal clinically important differences between groups.
2. The follow-up schedules for HoLEP patients in our study were not standardized, unlike the uniform 6-month follow-up implemented for the PAE cohort. This heterogeneity introduces a risk of temporal bias and could not be rectified through statistical tests, indicating a significant limitation in the study. Given the unequal follow-up durations, results were interpreted as cross-sectional comparisons rather than time-matched longitudinal outcomes. However, long-term studies indicate that key outcomes such as IPSS, Qmax, QoL, and erectile function stabilize after the first year post-HoLEP and remain durable up to 10 years in about 75% of cases [24].
  Likewise, PAE has demonstrated sustained symptom relief and low complication rates over 5–6 years [25]. These findings support our assumption that temporal variations in follow-up may have limited impact on the observed outcomes. Also, recent long-term studies have demonstrated that the outcomes of PAE remain stable over time, indicating that meaningful comparisons can still be made despite differences in follow-up intervals [15]. However, as the HoLEP cohort had substantially longer follow-up, whereas PAE outcomes were assessed at six months, the full therapeutic effect of PAE may not yet have been captured, potentially leading to a slight overestimation of the relative HoLEP benefit. Still, future studies should employ harmonized follow-up intervals to eliminate potential bias.
3. There is a variability in surgical experience. Since PAE is a relatively new procedure, the learning curve is a major factor in determining success rates and complications. Variations in operator expertise can critically influence the results of both interventions. In our PAE group, some procedures occurred during the early phase of the interventional radiologists’ learning curve, which could have affected technical success and complication rates. A study of 296 consecutive PAE procedures by radiologists without prior experience demonstrated that technical efficiency improved markedly after approximately 73–78 cases, confirming that early-stage operator inexperience diminishes with experience [26]. For HoLEP, outcomes have been shown to become consistent after approximately 40 procedures, depending on surgeon experience and institutional case volume. In the present study, the individual experience of the HoLEP surgeons was not documented in detail and therefore could not be directly compared with that of the interventional radiologists. However, as PAE was still a relatively new technique at the time of data acquisition, it is likely that the interventional radiologists were at an earlier stage of their learning curve. Conversely, the HoLEP surgeons may have had greater prior experience, although this assumption cannot be confirmed based on the available data.

Our study holds several strengths. To our knowledge this is the first study in the literature that compares functional outcomes and complications of PAE to HoLEP in a direct comparison, which allows a direct assessment. Further, the comparison is focused on procedures with an increasingly important urological practice, making the study’s findings highly relevant for both practitioners and patients.

The study evaluates several primary and secondary endpoints to allow a comprehensive evaluation of the investigated procedures. The systematic assessment of complications using the Clavien–Dindo score leads to an objective evaluation of the safety of both procedures.

Our results are relevant for daily practice, because they provide valuable information about the safety profile for the surgical risk as well as the symptom improvement and functional outcomes. Although the follow-up period for PAE is relatively short, the study provides long-term data for HoLEP. This offers valuable insight into the sustainability of treatment effects with HoLEP. In summary, this matched-pair analysis suggests that both PAE and HoLEP are effective and safe treatment options for patients with BPH, although our results should be interpreted with caution due to methodological limitations.

Future prospective studies with harmonized follow-up protocols are required to confirm these findings.

Still, the study provides clinicians with valuable guidance on which treatment to prefer based on patient profile and individual priorities. It supports patient-centered decision-making by highlighting the advantages and disadvantages of both treatments from various perspectives. When translating these findings into clinical practice, procedural selection should follow an individualized risk-benefit assessment.

PAE may be particularly considered in patients where a minimally invasive approach with a favorable safety profile and preservation of postoperative erectile function is desirable. HoLEP, in turn, showed larger numerical improvements in symptom scores and urinary flow in our cohort, although these between-group differences should be interpreted cautiously due to methodological limitations and the absence of consistent statistical significance across all endpoints.

## 5. Conclusions

Both PAE and HoLEP are effective treatment options for patients with LUTS due to BPH. Prostate size, comorbidities, symptom burden, and patient preference remain central determinants in shared decision-making. The present findings may help to inform the design of future investigations aimed at clarifying the specific clinical contexts in which each treatment offers the greatest benefit, ultimately supporting the development of future evidence-based guidelines.

## Figures and Tables

**Table 1:** Baseline characteristics of matched pairs; PSA, IIEF, IPSS, QoL, and Qmax. Data presented as median (IQR). *p*-value of <0.05 was considered statistically significant.

Characteristic	HoLEP	PAE	*p*
**No. pts (n)**	69	69	
**Age (y)**	66 (62–71)	67 (58–72)	0.8
**Prostate Volume (mL)**	75 (55–100)	61.8 (44.7–97.9)	0.050
**PSA (ng/mL)**	4.9 (2.4–9)	2.4 (1.5–5.4)	0.001
**IIEF**	18.5 (6.0–21)	23 (9.5–26)	0.002
**IPSS**	23 (16.5–27)	23 (17.5–26)	0.7
**QoL**	4 (4–5)	5 (4–5)	0.2
**Qmax (mL/s)**	10.6 (6.5–13.9)	10.0 (6–13)	0.4

**Table 2:** Pre- and postoperative functional results. Data presented as median (IQR). * Difference in preop minus postop.

Parameter	Preop	Postop	Difference	*p*
**HoLEP pts (n)**	69	69		
**IIEF**	18.5 (6.0–21)	15 (8–23)	−0.5 (−4–4)	0.9
**IPSS**	23 (16.5–27)	4 (1–8.5)	17 (10.5–22) *	<0.001
**QoL**	4 (4–5)	1 (0–2)	3 (2–4) *	<0.001
**Qmax (mL/s)**	10.6 (6.5–13.9)	25 (16.8–33.2)	14.9 (10.2–25.9)	<0.001
**PAE pts (n)**	69	69		
**IIEF**	23 (9.5–26)	24 (10–29.5)	0 (−1–5)	0.2
**IPSS**	23 (17.5–26)	8 (5–13)	13 (8–18.5) *	<0.001
**QoL**	5 (4–5)	2 (1–3)	3 (2–4) *	<0.001
**Qmax (mL/s)**	10 (6–13)	15.9 (7.7–19.7)	5.8 (2.2–9.2)	<0.001

**Table 3:** Characteristics of postoperative complications according to Clavien–Dindo Classification. Data presented as absolute value and frequency (%).

Grade/Complication	HoLEP	PAE	*p*
	(n = 69)	(n = 69)	
**Grade I (all)**	**9 (13.0%)**	**8 (11.6%)**	**1.0**
**-Grade I complications other than postop pain**	3 (4.3%)	8 (11.6%)	0.21
**Grade II (all)**	**5 (7.2%)**	**3 (4.3%)**	**0.7**
**Grade IIIa (all)**	**2 (2.9%)**	**0**	**0.5**
**Grade IV, V (all)**	**0**	**0**	

## Data Availability

The raw data supporting the conclusions of this article will be made available by the authors on request.

## Abbreviations

The following abbreviations are used in this manuscript:

BPH	Benign Prostatic Hyperplasia
BOO	Bladder Outlet Obstruction
DRE	Digital Rectal Examination
HoLEP	Holmium Laser Enucleation of the Prostate
IIEF	International Index of Erectile Function
IPSS	International Prostate Symptom Score
IQR	Interquartile Range
LUTS	Lower Urinary Tract Symptoms
PAE	Prostate Artery Embolization
PES	Postembolization Syndrome
PSA	Prostate-Specific Antigen
QoL	Quality of Life
Qmax	Maximum Urinary Flow Rate
SPSS	Statistical Package for the Social Sciences
TURP	Transurethral Resection of the Prostate
